# Genomic Complexity of *ccdc40* in *Xenopus* : Implications for CRISPR Targeting and Disease Modeling

**DOI:** 10.17912/micropub.biology.001596

**Published:** 2025-05-09

**Authors:** Takuya Nakayama, Saurabh Kulkarni

**Affiliations:** 1 Department of Biology, University of Virginia, Charlottesville, Virginia, United States; 2 Department of Cell Biology, University of Virginia, Charlottesville, Virginia, United States

## Abstract

Mutations in *CCDC40* cause primary ciliary dyskinesia in humans. To evaluate the pathogenicity of variants in *CCDC40* , we examined the genomic structure of this gene in *Xenopus tropicalis* , a diploid frog suitable as a model for genetic studies. We identified inconsistencies in the current *ccdc40* gene model and discovered two distinct *ccdc40* genes near the previously annotated locus. Surprisingly, *Xenopus* *laevis* , an allotetraploid species that typically has two homoeologs, contains only one homoeolog ( *ccdc40.S* ), making it a more suitable genetic model for studying *ccdc40* function and potentially expediting the functional characterization of CCDC40 variants linked to primary ciliary dyskinesia.

**Figure 1. The genomic region of Xenopus tropicalis ccdc40 genes:**
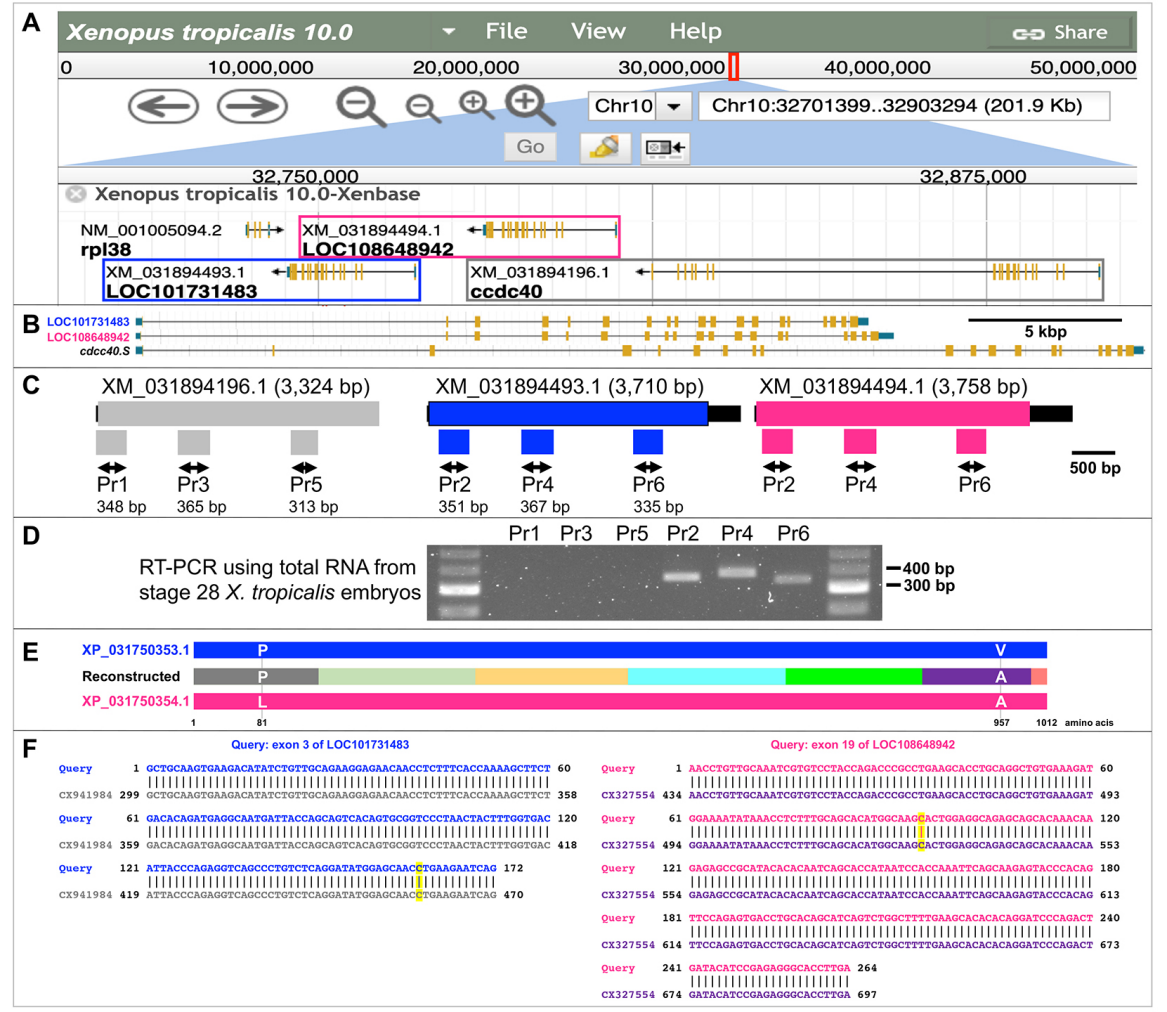
(A) A schematic representation of the *X. tropicalis* v.10.0 genome region on chromosome 10 (derived from Xenbase with their permission), spanning from 32,701,399 to 32,903,294, illustrates three gene models (each indicated by a square in blue, magenta, or gray) that contain published CRISPR target sequences for *ccdc40* . Note that all three genes are located on the minus strand. (B) A schematic comparison of *ccdc40* gene models (derived from Xenbase with their permission) among *X. tropicalis* LOC101731483 (24,053 bp; Chr10:32744235..32768287 [- strand]) (top), *X. tropicalis* LOC108648942 (24,872 bp; Chr10:32780961..32805832 [- strand]) (middle), and *X. laevis* *ccdc40.S* (33,142 bp; Chr9_10S:21823636..21856777 [- strand]) (bottom). The orientation of genes is shown in the direction from 5' (left) to 3' (right) for convenience and is drawn to scale. (C) A schematic representation of the cDNAs for each gene within the squares shown in (A) is presented and drawn to scale. Thick black lines indicate the full length of predicted cDNAs, while ORFs are depicted in colored boxes that match the square colors in (A) to demonstrate the relationship between cDNAs and genes. The predicted cDNA (XM_031894196.1) of the "former" *ccdc40* gene lacks the 3'-UTR. Beneath each cDNA, schematic representations of positions and amplicon sizes for each primer set are shown: Pr1, 3, 5 for XM_031894196.1, and Pr2, 4, 6 for XM_031894493.1 and XM_031894494.1. (D) The representative gel image depicts the RT-PCR results. (E) A schematic comparison of the protein sequence of XP_031750353.1 (top), the protein sequence reconstructed from seven ESTs (middle), and the protein sequence of XP_031750354.1 (bottom). Protein sequences reconstructed from different ESTs are distinguished by different colors. The gray (from CX941984) and purple (from CX327554) regions contain amino acids 81 and 957, respectively, showing single amino acid change. Other protein regions are identical among the three sequences. The figures are drawn to scale. (F) The left panel displays the alignment of exon 3 from LOC101731483 and CX941984, while the right panel shows the alignment of exon 19 from LOC108648942 and CX327554. Yellow-highlighted portions indicate SNP locations. Only aligned sequences are shown for ESTs. The color code matches (E).

## Description

CCDC40 is a coiled-coil domain-containing protein 40 that is essential for establishing the left-right axis and proper motile cilia function in the airway. Consistently, loss-of-function mutations in *CCDC40* have been shown to cause defects in motile cilia function in humans, mice, zebrafish, and *Xenopus tropicalis* (Becker-Heck et al., 2011; Bhattacharya et al., 2015), leading to primary ciliary dyskinesia (PCD) in humans (DOID:9562; MIM:244400).

The emergence of gene-editing technologies, particularly Clustered Regularly Interspaced Short Palindromic Repeats/CRISPR-associated protein 9 (CRISPR/Cas9), has revolutionized genome manipulation methods. CRISPR/Cas9 has been established in both *Xenopus tropicalis* (Blitz et al., 2013; Nakayama et al., 2013) and *Xenopus laevis* (Wang et al., 2015), demonstrating their suitability as models for developmental genetics alongside classical embryology research. Due to its simpler diploid genome (Hellsten et al., 2010), *X. tropicalis* holds promise for modeling human genetic diseases (Hwang et al., 2019; Willsey et al., 2024), especially ciliopathies (Rao & Kulkarni, 2021), in contrast to the allotetraploid genome of *X. laevis* (Session et al., 2016).

During our research to investigate the pathogenicity of variants of unknown significance (VUS) in the *ccdc40* gene (GeneID:100486955) affecting motile cilia function in *X. tropicalis* , we used CRISPR targeting and encountered significant challenges. Specifically, identifying a unique CRISPR/Cas9 target site without potential off-target effects was difficult (see (Blitz & Nakayama, 2022) for the strategy). Upon examination, we found that the previously published CRISPR/Cas9 target sequence, including its PAM site (Bhattacharya et al., 2015), appeared four times throughout the genome, raising concerns about off-target activity. Further complicating our analysis, the forward and reverse primers previously used for genotyping, designed to amplify an approximately 200 bp region containing the CRISPR/Cas9 target site, also matched four distinct genomic locations. Additional investigation using Xenbase (Fisher et al., 2023) confirmed these four genomic matches: two within the *ccdc40* gene itself (referred to as the "former" *ccdc40* , detailed further below), one in the gene LOC101731483 (GeneID:101731483), and one in LOC108648942 (GeneID:108648942) ( Figure 1A ). This suggests that the published genotyping results (Bhattacharya et al., 2015) must be mixed with data from four different genomic regions.

In *X. laevis* , despite its allotetraploid genome, the *ccdc40* gene exists as a singleton found only in the S genome ( *ccdc40.S* [GeneID:108702701], XM_018238324.2; Xenbase, X. laevis 10.0). The predicted structure of *ccdc40.S* comprises 19 exons ( Figure 1B ). The genes of *X. tropicalis* LOC108648942 and LOC101731483 also contain 19 exons ( Figure 1B ), while the "former" *ccdc40* gene has 22 exons. Notably, this "former" *ccdc40* gene features six exons that are repeated. For example, especially "100% identity" between exon 2 and exon 16, exon 6 and exon 20, and exon 7 and exon 21 was observed. Moreover, the previously published CRISPR target site (Bhattacharya et al., 2015) appears twice within this gene—in exon 4 and exon 18—exhibiting 96.81% identity. Consequently, exons from 16 to 21 appear to be duplicated versions of exons 2 to 7, showing 96.81% to 100% identity between corresponding exons.

We decided to perform RT-PCR analysis to evaluate gene expression further and detect transcripts from these genes. Due to the high similarity between LOC108648942 and LOC101731483 (99.75% identity based on their predicted cDNAs, XM_031894494.1 and XM_031894493.1 from NCBI, (Sayers et al., 2024)), distinguishing between their transcripts by RT-PCR proved challenging. However, it was possible to design primers specific to the "former" *ccdc40* (predicted) mRNA sequence (XM_031894196.1), which is 98.92 and 98.96% identical to XM_031894494.1 and XM_031894493.1, respectively. We selected three similar regions from the 5' to 3' ends of each predicted mRNA for amplification ( Figure 1C ). Our RT-PCR results ( Figure 1D ) indicate that transcripts from the primer regions matching LOC108648942 and LOC101731483 were detectable, while transcripts from the "former" *ccdc40* gene were not detectable at developmental stage 28, a critical period when multiciliated cells (MCCs) containing motile cilia mature (Kulkarni et al., 2021; Kulkarni et al., 2018).

To further confirm the expression of LOC108648942 and LOC101731483, we performed a BLAST search in the NCBI database for Expressed Sequence Tags (ESTs) matching their predicted cDNAs. We identified multiple ESTs (e.g., CX941984, AL632867, CN086103, CX937961, DT449052, CX327554, EL834361) and successfully reconstructed a complete potential mRNA sequence corresponding to LOC108648942 and LOC101731483 from 5' to 3'. Although we also could identify some ESTs that overlapped with all three predicted cDNAs due to high similarity, we could not reconstruct a complete mRNA sequence corresponding to the "former" *ccdc40* (XM_031894196.1). The ESTs we identified were derived from various tissues, including tadpole brains and spinal cords, adult spleen and testes, and whole embryos, suggesting widespread expression of LOC108648942 and LOC101731483.

The reconstructed mRNA encodes 1,012 amino acids, which is the same length as LOC108648942 and LOC101731483, corresponding to XP_031750354.1 and XP_031750353.1, with an amino acid identity of 99.90% ( Figure 1E ). In contrast, the "former" *ccdc40* (XP_031750056.1) encodes 1,078 amino acids, displaying a slightly lower identity of 98.96% compared to the reconstructed sequence. When compared to Ccdc40.S from *X. laevis* (XP_018093813.1, 1,045 amino acids), LOC108648942 and LOC101731483 exhibit approximately 85% identity, while the "former" *ccdc40* shows around 82% identity. Considering gene structures, mRNA expression data, and protein sequence similarities, we suggest that the "former" *ccdc40* gene is likely not functional. Instead, we propose that LOC108648942 and LOC101731483 represent the authentic *ccdc40* genes in the *X. tropicalis* genome, although further evidence is needed to confirm this conclusively.

When comparing LOC108648942 and LOC101731483 (XM_031894494.1/XM_031894493.1), several single-nucleotide polymorphisms (SNPs) were identified in the protein-coding regions and untranslated regions (UTRs). Some SNPs resulted in silent mutations, while others caused missense mutations. We focused on missense mutations to clearly distinguish the two genes. LOC108648942/XP_031750354.1 encodes the amino acids L81 and A957, whereas LOC101731483/XP_031750353.1 encodes P81 and V957 at the corresponding positions ( Figure 1E ). EST analysis further supported transcription from both genes; one EST (CX941984) contained a SNP corresponding to LOC101731483 in exon 3 encoding P81, and another EST (CX327554) corresponded to LOC108648942 in exon 19 encoding A957 ( Figure 1E, F). Given these observations and their closer similarity to *X. laevis* Ccdc40.S (that contains L81 and A990 at the corresponding sites), we propose renaming LOC108648942 as *X. tropicalis ccdc40* and LOC101731483 as *ccdc40.2* , following *Xenopus* gene nomenclature.

The Harland group (Grammer et al., 2005) reported a naturally occurring background mutant known as " *grinch* " in *X.* *tropicalis* Nigerian strain colonies from various laboratories, including the Grainger lab. These mutants exhibit significant ventral edema. Subsequently, the Khokha lab (del Viso et al., 2012) identified ciliary defects in this mutant, linking them to multiple mutations in the " *ccdc40* " gene based on genome data v4.1 and v7.1. They predicted that the gene had 18 exons, in contrast to the 19 exons we identified based on genome version 10.0 ( Figure 1B ); however, they correctly predicted the encoded protein length to be 1,012 amino acids.

Notably, although the phenotype observed by Grammer et al. (2005) suggested simple Mendelian inheritance (a single recessive allele resulting in a 25% mutant phenotype), the mutant cDNAs analyzed by del Viso et al. (2012) revealed multiple mutations (Figure 4b in del Viso et al., 2012), indicating the presence of more than one mutant allele. Given our findings presented here, some of their results might be explained by the existence of two separate *ccdc40* genes. Therefore, a detailed re-examination of the *grinch* mutation locus would be valuable in the future, explicitly considering the two-gene scenario. Additionally, we noted that the mutant cDNAs and predicted proteins described by del Viso et al. (2012; Supplementary Figure 5) exhibit amino acids representative of both genes (L81 from LOC108648942/ *ccdc40* and P81 from LOC101731483/ *ccdc40.2* ), again supporting transcriptional activity of both genes.

The loss of the *ccdc40* gene from the L genome of *X. laevis* and the genomic complexity (three similar genes and naturally occurring mutations such as *grinch* ) in the *ccdc40* -containing region of *X. tropicalis* may indicate that this genomic region is prone to mutation in *Xenopus* species. It would thus be insightful to investigate this region across other *Xenopus* species and additional *X. tropicalis* strains beyond the Nigerian strain.

In summary, the existence of two *ccdc40* genes ( *ccdc40* and *ccdc40.2* ) in *X. tropicalis* complicates genotyping and CRISPR targeting. Therefore, we suggest that *X. laevis* , which has only one *ccdc40.S* gene, offers a simpler and more suitable genetic model for studying Ccdc40 function.

## Methods

**Frogs**

*X. tropicalis* colony is from our in-house breeding stocks at the University of Virginia, which are not highly inbred but originated from the same ancestors as the Nigerian inbred line used for genome sequencing (Hellsten et al., 2010). Frogs ( *Xenopus tropicalis* ) were bred and housed in a vivarium using protocols (ACUC# 4295, 1472) approved by the University of Virginia Institutional Animal Care and Use Committee (IACUC). Embryos needed for experiments were generated using *in vitro* fertilization as described before (Nakayama & Grainger, 2023; Rao et al., 2025). Briefly, the testes from male frogs were crushed in 1x MBS (pH 7.4-7.5) with 0.1-0.2% BSA and added to eggs obtained from the female frogs. After 1-3 minutes of incubation, freshly made 0.1x MBS (pH 7.4-7.5) was added, and the eggs were incubated for 10 more minutes till contraction of the animal pole of the eggs was visible. The jelly coat was removed using 2-3% cysteine in 0.1 x MBS solution (pH 7.8-8.0) for 3-5 minutes (until the jelly coat was removed).

**RNA extraction and cDNA synthesis (for RT-PCR)**

Five embryos at stage 28 (staged according to (Nieuwkoop, 1994)) were pooled and subjected to total RNA extraction using Quick-RNA ^TM^ Miniprep Kit (Zymo Research), following the manufacturer's instructions. 5 ng of the resultant total RNA was used to make cDNA using Verso cDNA Synthesis Kit (Thermo Scientific™), following the manufacturer's instructions

**PCR**

PCR reactions used EmeraldAmp® GT PCR Master Mix (Takara Bio), following the manufacturer's instructions. Each 12.5 μl reaction contained 0.5 μl cDNA and specific primer sets (Figure 1). For PCR, after denaturing the PCR mixture at 94°C for 5 min, the condition of one PCR cycle was as follows: denaturing at 94°C for 20 sec; annealing (see below for temperature) for 20 sec; extension at 68°C for 12 sec. Touchdown PCR (Korbie & Mattick, 2008) was performed, namely, by gradually reducing the annealing temperature (initially set at 65°C) in 0.5°C increments per cycle for 13 cycles, followed by additional cycles at a constant annealing temperature of 58°C. The number of additional cycles required for each primer set was determined for each primer set (see Table 1) by trial and error, i.e., 26 cycles for Pr Set1 and 2; 19 cycles for Pr Set 3 and 4; 17 cycles for Pr Set5 and 6. Subsequently, 5 μl of the PCR product for each primer set was loaded for electrophoresis using 2% agarose gel by the standard method.

***In silico* assay**

Homology searches for short sequences like CRISPR target sites or primer were conducted using GGGenome (https://gggenome.dbcls.jp/). Gene structure and BLAST searches were performed with Xenbase (http://www.xenbase.org/, RRID:SCR_003280) and NCBI databases (https://www.ncbi.nlm.nih.gov/). PCR primers were designed using Primer3 (v. 0.4.0) (https://bioinfo.ut.ee/primer3-0.4.0/).

## Reagents

**Table 1. RT-PCR primers**

**Table:** 

Primers: sequence (5' to 3')		*Target regions in XM_031894196.1*	*Target regions inXM_031894493.1*	*Target regions inXM_031894494.1*
Pr Set 1	Pr1_5Pr: CCCATCTCACCCTGCACTAT * Pr1_3Pr: ATGACCCCACCACTCCTGA	8-355(348 bp)	N/A	N/A
Pr Set 3	Pr3_5Pr: GAAAAAAAAACAGGATATTTTTGTGG Pr3_3Pr; CTGCACGGTTCAACATTAGC	972-1,336(365 bp)	N/A	N/A
Pr Set 5	Pr5_5Pr: TGCAGGAAAATCTACGAAACC ** Pr5_3Pr: CCTGTTTCACCTGATTCTTCAA	2,306-2,618(313 bp)	N/A	N/A
Pr Set 2	Pr2_5Pr: CCCATCTCACCCTGCACTAT * Pr2_3Pr: CCACCACTCCTGTTTCACCT	N/A	133-483(351 bp)	83-433(351 bp)
Pr Set 4	Pr4_5Pr: AAAAAAGCAGGATATTTTTGTGG Pr4_3Pr: TGCACGGTTCAACATGAAGT	N/A	1,115-1,481(367 bp)	1,065-1,431(367 bp)
Pr Set 6	Pr6_5Pr: TGCAGGAAAATCTACGAAACC ** Pr6_3Pr: CCTACGAAAGTCACTGACGGTTA	N/A	2,452-2,786(335 bp)	2,402-2,736(335 bp)

*, commonly used for Pr Set1 and 2; **, commonly used for Pr Set5 and 6.
